# Man and His Enemies

**Published:** 1889-03-09

**Authors:** 


### The Doctor's Pulpit.

### MAN AND HIS ENEMIES.

There is an unbroken unanimity of opinion that man is the greatest of all the creatures which inhabit this sphere. Every freshman knows that, according to all the philosophers, "On earth there is nothing great but man." That is not very civil to the crustaceans of whom Huxley is so much enamoured, nor to the microbes, admired and loved by Koch. But if the crustaceans or the microbes wish to establish their claim to equality, they must bestir themselves to better purpose. As yet they have made no attempt at producing a literature; they have not invented a steam engine, or even a lucifer match. The monkeys have obtained a kind of academic recognition at the hands of certain illuminati; but it is to be feared that that is purely academic, and by no means to be presumed upon. For although in high philosophic discourse the Negro and the Aztec are held to be much in advance even of the monkeys, quite forward in fact, on the lives of elevated development, yet if individual Negroes or Aztecs were to propose to dine with the philosophers, or still more, to marry their daughters, it is to be feared that language not strictly academic would fall unwelcome on their ears. If then in actual practice the negro is held to be so inferior as to be beyond the pale of possible equality, the monkeys, in spite of the civil speeches of their friends the philosophers, had better abandon all hope of anything like a practical recognition of their high and human virtues and capabilities. The parson will address his poorer hearers as "brethren" from the pulpit, but in the street Hodge knows how to keep his distance. So with the philosophers and the monkeys. The term "Anthropoid" is very civil in the lecture-hall, but when the "anthropous" one claims his rights in the drawing room, he is speedily relegated to his cage and his bed of straw.

Man then is alone great. We may lay that flattering unction to our souls without fear of contradiction from philosopher or priest. It is very true that Carlyle, in one of his most vitriolic moments, declared that there were four millions of people in London, mostly fools. But even he at a more rational period generously allowed man to be truly great. Nay, more, in discoursing sympathetically of the miseries man endures, he affirmed that those very miseries are solely due to his greatness. As a matter of fact, no individual man doubts for a moment that he is more important in the scale of being than either the amœba, or the crustacean, or the amphibian, or the quadrupedal or quadrumanal mammal, or, indeed, than all of them put together. If this earth was not originally designed for man, it is quite clear that he has by some means gained the first and the ruling place in it. The writer of the book of Genesis calls him to "dominion over the fish of the sea, the fowls of the air, and all the creeping and moving things on the face of the earth." Whether that was a call of authority or not, here is the undoubted fact, that man is the earthly deity and ruler of all the other creatures that inhabit his own particular world.

All living things have their enemies. The sparrow sitting on the ridge of the house in winter ruffles his feathers about him to keep the biting frosty air a quarter of an inch further away from his shivering little body. The crocus peers out of the ground in the cold spring as if he knew that the keen March wind carried death in his piercing blasts. The rabbit nibbles his grass on the sandy heath, with ears alert and eyes in all directions, ready to bolt and scuttle to the safety of his hole at the first evidence of strange sights or sounds. Even the child, lying awake in the darkness, covers his head with the bed-clothes to hide himself from a danger which he does not understand. Every creature has its environment, and that environment means things helpful and things injurious, enemies as well as friends. Man is no exception. At every period of his life he is in the midst of dangers. They haunt him from the cradle to the grave; nay, even before the cradle, and it may be after the grave. The moralist sees one class of dangers, the economist another, the physician another, and the physiologist another. The man, if he included in himself, as he would if he were a typical man, both moralist, economist, physician, and physiologist, would see every class. Seeing that he has to fight every class, and in his own person to vanquish or be vanquished by one or all of them, it is incumbent upon him to see and understand them all, and to take them all into account in his plan of life. If it be said that this is too great a burden to lay upon any man, the answer is easy: Nature or Providence has laid it upon him; and he is compelled by a necessity which knows no shadow of yielding, to bear it or sink under it. Philosophers have discovered that man is great, and is called to great duties and destinies. Every man finds out for himself that great duties and destinies lie before him, and urge themselves constantly upon his will. But if he does not find in himself the valiant greatness which accepts the responsibility it would have been better for him if he had never been born.

What is the use of quarrelling with facts? What is the wisdom of denying the inevitable? An iron necessity is around us and a free will within. It is for us to make the best of ourselves in the world and among the circumstances in which we find ourselves. But so far as we can see the necessity has its limits; and it too has been fixed so as to secure the highest development and advantage of the human creature. Enemies we are surrounded by, numerous, subtle, powerful; and undoubtedly many human creatures suffer what appears to be final defeat. But there is in every sane normal man that which makes for success and victory; and, in the present writer at least, the conviction is immovable that in the world's battle no man can finally and irrevocably fail, except by his own will and his own unworthiness.

The battle to-day is a physiological battle; but physiological in the largest not in the meanest sense. Physiology is the science of structure and function; not of structure alone, nor of the lower functions, but of every function of every part of the organism. Can that man be called a physiologist who labours to understand the function of the liver, but takes no account of that of the brain? Can he be called a man of science who studies the reflex functions of the brain and gives no heed to its reasoning powers? Is he a philosopher who recognises its reasoning powers but decries or despises its moral and religious instincts? Man must be studied as man, in his entirety. His environment must be taken in its breadth and completeness. All the adverse forces which act upon him, whether from within or from without, must be taken into review in any adequate survey of "man and his enemies." Such a subject is in itself profoundly important, and ought to be as interesting as it is great and weighty. It is not for philosophers to claim a monopoly of it, or for plain people to admit the claim. If philosophers were compelled to bear all the responsibility of every man's life, and all the consequences of his stupidity, or his indolence, or his failure, then, indeed, they might be left in possession of the field of study. But inasmuch as each man must fight his own life battle for himself, and wil l reap for himself the rewards of victory, or must accept for himself the bitter consequences of defeat, it is incumbent upon every one to open his eyes, to look around him, to arm himself for the conflict, and to fight with the best skill and valour of which he is capable. Conflict to the weak and the cowardly is a thing to be dreaded and avoided by all possible means; but to the valiant, the heroic, and the worthy it is stimulus and delight. The base man meanly asks "Is life worth living?" The honourable marches forth to the conflict and declares that the battle itself with all its turmoil and danger is as the very breath of morning to his nostrils. Physiology, properly understood, is a soldier's handbook which teaches every man so to use his weapons and his opportunities throughout life as finally to secure for himself the fruits of victory.

(*To be continued.*)

